# The role of G-CSF in recurrent implantation failure: A randomized double blind placebo control trial

**Published:** 2016-12

**Authors:** Fatemeh Davari-tanha, Ensieh Shahrokh Tehraninejad, Mohadese Ghazi, Zahra Shahraki

**Affiliations:** 1 *Department of Reproductive Endocrinology, Women Hospital, Vali-e-Asr Health Reproductive Center, Tehran University of Medical Sciences, Tehran, Iran.*; 2 *Department of Reproductive Endocrinology, Vali-e-Asr Hospital, Vali-e-Asr Health Reproductive Center, Tehran University of Medical Sciences, Tehran, Iran.*

**Keywords:** *Implantation*, *Failure*, *G-CSF*, *Intrauterine*, *Pregnancy rate*

## Abstract

**Background:**

Recurrent implantation failure (RIF) is the absence of implantation after three consecutive In Vitro Fertilization (IVF) cycles with transferring at least four good quality embryos in a minimum of three fresh or frozen cycles in a woman under 40 years. The definition and management of RIF is under constant scrutiny.

**Objective:**

To investigate the effects of Granulocyte colony stimulating factor (G-CSF) on RIF, pregnancy rate, abortion rate and implantation rates.

**Materials and Methods:**

A double blind placebo controlled randomized trial was conducted at two tertiary university based hospitals. One hundred patients with the history of RIF from December 2011 until January 2014 were recruited in the study. G-CSF 300µg/1ml was administered at the day of oocyte puncture or day of progesterone administration of FET cycle. Forty patients were recruited at G-CSF group, 40 in saline and 20 in placebo group.

**Results:**

The mean age for whole study group was 35.3±4.2 yrs (G-CSF 35.5±4.32, saline 35.3±3.98, placebo 35.4±4.01, respectively). Seventeen patients had a positive pregnancy test after embryo transfer [10 (25%) in G-CSF; 5 (12.5%) in saline; and 2 (10%) in placebo group]. The mean of abortion rates was 17.6% (3), two of them in G-CSF, one in saline group. The implantation rate was 12.3% in G-CSF, 6.1% in saline and 4.7% in placebo group.

**Conclusion:**

G-CSF may increase chemical pregnancy and implantation rate in patients with recurrent implantation failure but clinical pregnancy rate and abortion rate was unaffected.

## Introduction

Recurrent implantation failure (RIF) is defined as failure to achieve pregnancy after in vitro fertilization (IVF) or intracytoplasmic sperm injection, after transferring at least four good quality embryos in at least three fresh or frozen cycles in women younger than forty years old (1). Repetitive implantation failure is an iatrogenic event that is the consequence of embryo or uterine factors (2, 3). RIF is different from IVF failure. IVF failure is failure to achieve pregnancy due to poor responder to ovarian induction, absence of good quality embryo, advanced maternal age and finally uterine factors (4, 5).

Nowadays RIF is defined basically as implantation failure due to uterine factors. Actually RIF is a subgroup of IVF failure patients who have good quality embryo and age <40 but have failure to achieve pregnancy (5). Mullerian anomalies like septate uterus can change endometrial receptivity not only due to disturbance of uterine cavity but also to the inadequate blood supply to the septum (6). Abortion rate after IVF in septate uterus was reported 80% but after hysteroscopic resection of septum decreased to 30%. Submucosal myoma increases uterine contractility and changes cytokine profile; also it leads to abnormal vascularization and chronic endometiritis. These women have decreased implantation rate. Resection of submucosal myoma hysteroscopically results to double clinical pregnancy rates (7). Also resection of endometrial polyps resulted to more clinical pregnancy rate in Intra Uterine insemination (IUI) cycles (8). 

Besides the submucosal myoma and polyp, intrauterine adhesion either after dilatation & curettage (D&C) in gravid uterus for abortion or after intrauterine infection in nongravid uterus may interfere with successful implantation (9). On the other hand adenomyosis which affects the junctional zone of uterus is more hazardous for implantation than intramural myoma which is far from implantation site (10). Moreover hydrosalpinx removal results to improve pregnancy outcome after IVF (16% vs. 28.6% live birth rate) (11,12). 

However the immunological factors are considered critical for embryo implantation, there are much conflicting evidence on the value of immunological treatment in patients with RIF (13, 14). G-CSF is a hematopoietic specific cytokine produced by bone marrow cells, stromal cells, fibroblasts and macrophages. G-CSF increases phagocytosis and oxidative process which is necessary for implantation (15). Some of nonhematopoetic cell types, including endothelial, placenta, trophoblastic and granolousa luteina cells contain G-CSF receptor (14). Moreover GCSF appears to affect endometrial expression of genes critical for the implantation process, such as endometrial vascular remodeling, local immune modulation and cellular adhesions mechanisms (15). 

G-CSF contributes to successful reproduction by increasing implantation and promoting endometrial thickness. Nevertheless many cases of RIF remain unexplained and several etiological factors including immune dysfunction or alloimmune response are proposed; in animal models, G-CSF showed a marked anti abortive activity (16). G-CSF play roles in increasing endometrial thickness and decreasing recurrent abortion (6, 17-19).

The aim of the present study was the evaluation of G-CSF effects on patients with RIF regarding to pregnancy, abortion and implantation rates.

## Materials and methods

**Figure 1:**
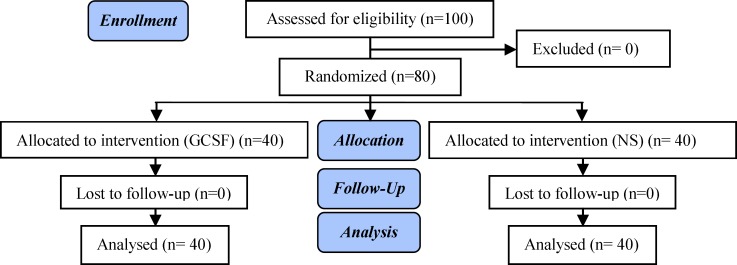
Consort flowchart.

In a randomized double blind placebo controlled clinical trial 100 patients with recurrent implantation failure were included from two tertiary university based hospitals of Tehran University of Medical Sciences from December 2011 until January 2014 in this study. The study was approved by the ethical committee of Tehran University of Medical Sciences with the project code of 14967. All eligible patients signed informed consent before participation in the study. This study was a three-arm randomized clinical trial. Randomization was performed by a computer-generated randomization block table. Randomization cards were offered to the patients by a nurse who was blinded to the study groups. Patients and clinician were blinded regarding the study groups (Figure 1).

Embryo transfer was done as frozen and fresh cycles. Nine patients underwent FET cycle (four patients in G-CSF; 3 patients in saline and two in placebo group) and 91 patients underwent fresh cycle. Inclusion criteria were all patients with RIF under the age of 40 yr old (mean age= 35.3±4.2 yr). RIF was defined as history of three times implantation failure when there was history of transferring at least four good quality embryos without uterine or thrombophilic factors. Women with history of renal disease, sickle cell disease or malignancy or sensitivity of G-CSF were excluded from study. Gonal-F/HMG was prescribed at a dosage of 150-225 U per day on the second day of menstruation cycle. The ovarian response was evaluated by transvaginal sonography and the need for additional dose was determined according it. 

Recombinant hCG was injected when there were at least 3 follicles above 18 mm, and oocyte retrieval was performed 36 hr after injection by transvaginal ultrasound guidance. The G-CSF used was Nupogen (300 µg/ml, Filgrastim; Amgen). At the time of oocyte retrieval one ml of G-CSF or normal saline was administered by a Trans cervical Cook catheter for embryo transfer slowly into uterine cavity. For placebo group a catheter pass through the cervix without any injection. In FET cycle intervention was scheduled at the day of starting progesterone. Chemical pregnancy was defined as positive β-hCG two weeks after embryo transfer. Clinical pregnancy was assessed by visualizing gestational sac at transvaginal sonogram three weeks after embryo transfer. 

Implantation rates were defined as number of gestational sac four weeks after embryo transfer based on the number of embryos transferred. The good quality embryo at day three was an 8 cells embryo with <15% fragmentation or at day five an expanded blastocysts with at least grade B trophoectoderm and inner cell mass.

**Statistical analysis**

All analysis conducted by Statistical Package for the Social Sciences, version 16.0, SPSS Inc, Chicago, Illinois, USA (SPSS 16). For continuous variables, statistical significance was assessed by the use of the two tailed student’s t-test for unpaired data. Fisher exact test and ^2^ were used when appropriate for discontinuous variables. p<0.05 was considered significant.

## Results

The demographic data and baseline characteristics of patients are shown in table I. No differences were found in three groups of patients for the women’s age, number of pervious IVF failures, Body Mass Index (BMI), and the number of good quality embryo transfer. The mean follicle stimulating hormone (FSH) level was 7.36±3.64 (G-CSF 7.98±4.21, saline 7.22±2.98, and placebo 7.41±2.51). The mean of endometrial thickness at the day of hCG trigger was 10.23±2.51 mm (G-CSF 9.98±3.61, saline 10.35±1.45, and placebo 10.01±2.59).

There were no significant differences in three groups. Seventeen patients had a positive β-hCG titer after embryo transfer (10 in G-CSF group, 5 in saline and 2 in placebo group). Fourteen patients established clinical pregnancy: in G-CSF 25% (8 of 40 patients); in saline group 12.5% (5 of 40 patients) and in placebo group 10% (2 of 20 patients). The difference of chemical pregnancy was significant for G-CSF group comparing to saline and placebo group (p<0.04) but the difference between saline and placebo group was not significant (p<0.15). 

There were three spontaneous abortion, two out of ten in G-CSF group and one out of five in saline group (p=0.06). Also 210 embryos were transferred during study. The implantation rate was 12.3% (10 of 81) in G-CSF; 6.1% (5 of 87) in saline and 4.7% (2 of 42) in placebo group. The implantation rate was statistically different for G-CSF regarding saline and placebo group (p=0.04). The quality of embryo in three groups was not statistically different but there were more grade A embryos in placebo group.

**Table I:** Demographic characteristics of participants in three groups (G-CSF, saline, placebo) of RIF patients

**Variable**	**G-CSF**	**Saline**	**Placebo**	**p-value**
Age (y)	35.5 ± 4.32	35.3 ± 3.98	35.4 ± 4.01	0.33
BMI (kg/m2)	25.2 ± 1.8	23.9 ± 2.01	24.8 ± 1.3	0.41
Number of IVF failure (n)	3.5 ± 2.1	4.2 ± 1.5	3.9 ± 1.06	0.34
Number of good quality ET (n)	2.91 ± 0.85	2.85 ± 0.67	2.94 ± 0.53	0.54
FSH (mu/ml)	7.98 ± 4.21	7.22 ± 2.98	7.41 ± 2.51	0.61
3^rd^ day Esteradiol (µg/ml)	33.51 ± 4.65	35.41 ± 2.51	34.33 ± 3.22	0.32

Data presented as mean±SD.

BMI: Body Mass Index

IVF: In Vitro Fertilization

ET: Embryo Transfer

**Table II:** Outcome of cycles in three groups (G-CSF, saline, placebo) of RIF patients

**Variables **	**G-CSF (n=40) **	**Saline (n=40) **	**Placebo (n=20) **	**p-value**
Abortions	2 (20%)	1 (25%)	0	0.06
Chemical pregnancy	10 (25%)	5 (12.5%)	2 (10%)	0.04
Clinical pregnancy	8/10 (80%)	4/5 (80%)	2/2 (100%)	0.51
Implantation rate	10/81 (12.3%)	5/87 (6.1%)	2/42 (4.7%)	0.04
Fresh embryo transfer	66 (81.4%)	77 (88.5%)	35 (83.3%)	--
FET transfer	15 (18.5%)	9 (11.49%)	7 (16.6%)	--

Data presented as n (%).

GCSF: Granulocyte Colony Stimulating Factor

FET: Frozen Embryo Transfer

## Discussion

The results of this trial suggests that intrauterine perfusion of G-CSF in RIF can increase chemical pregnancy and implantation rates however the effects on clinical pregnancy rate and abortion rate need to be evaluated. RIF may be the consequence of embryo or uterine factors. Uterine pathology like myoma, polyp, adenomyosis and adhesion can cause implantation failure; however patients with RIF have vigorously assessed for these pathologic condition and many of them have history of laparoscopic or hysteroscopic correction and evaluation of these disorders (20, 21). 

However 40% of patients with RIF have some unrecognized lesions in endometrial cavity; in the rest there is no detectable pathology at uterine cavity (22). All eligible patients in present study have been evaluated for uterine pathology by hysteroscopy or synchronous laparoscopy. The embryo attaches to the luminal surface of the endometrium, then migrates via the luminal epithelium and invades into deep layer of endometrium which leads to implantation. Desidualization of endometrial cells is a differentiation process and it is crucial for implantation of pregnancy (2). G-CSF stimulates cellular differentiation of hemopoietic progenitor cells (23-26). The present study showed that G-CSF increases the chemical pregnancy and implantation rate in women with RIF.

In a randomized study in women with recurrent abortion 82.9% of women who were treated with G-CSF subcutaneously delivered healthy infants (p=0.0061, OR=5.1; 95% CI 1.5-18.4) (27). In a randomized trial endometrial injury resulted to a significant improvement (nearly development) in the implantation and clinical pregnancy and live birth rate (27.7%, 66.7% and 48.9% respectively) compared with control subjects who did not have endometrial biopsies (28,29). G-CSF administration appears to be associated with an increase in regulatory T cells and dendritic cells and appears to influence endometrial expression of genes which have cardinal role in implantation process (30-33).

Santjohander in 2013 showed that G-CSF in patients with recurrent miscarriage leads to better reproductive results regarding to placebo. Pregnancy rate of 47% and live birth rate of 32% was reported in G-CSF group but pregnancy rate of 27% (p=0.016) and live birth rate of 14% (p=0.006) was reported in placebo group (34). Gleicher *et al* reported four patients with thin endometrium whom were treated with intrauterine G-CSF, except one with ectopic pregnancy, three other patients had ongoing pregnancy at the time of study report (35).

Gleicher *et al* reported 21 patients with thin endometrium who were treated with intrauterine G-CSF. The mean age of the patients was 40.5±6.5 years and 76.2% of them had poor ovarian response. They had history of IVF failure (2±2.1) cycles and also history of cycle cancellation due to thin endometrium (36). Endometrial thickness significantly increased and pregnancy rate was 19%. They showed that G-CSF can increases endometrial thickness, but the sample size was small and there was no control group. At present study there were case group (G-CSF), control group (saline) and placebo group (without intervention) and this study showed that chemical pregnancy rates were significantly different in G-CSF group regarding to saline or placebo group.

Pregnancy outcome in 37 patients with thin endometrium (<7mm) was evaluated by Kunicki. G-CSF results in improvement of endometrial thickness in both group (patients who become pregnant and patients who did not) (37). The pregnancy rate in this study was 18.9%.

Li *et al* reported a cohort of 59 patients in FET cycle who intrauterine G-CSF during endometrial preparation were administered. Implantation rate and pregnancy rate was not significantly different (38). However the study was retrospective and the dose of G-CSF was 100µg. In clinical trial of fifteen patients with history of thin endometrium who had history of cycle cancellation due to thin endometrium, G-CSF intrauterine could increase endometrial thickness and 3 out of 15 patients became pregnant. The pregnancy rate was 19% (39). Thin endometrium was resistant to other treatment, like high dose estrogen, sildnafiel, Asprin or even vit-E. 

Barad *et al* reported that G-CSF in patients with normal endometrium and old age had no effects in pregnancy and implantation rates (40). They evaluated 141 unselected women without history of renal disease, sickle cell or malignancy who were undergone IVF. Seventy three of them received G-CSF and 68 received placebo. The increase in endometrial thickness was not statistically different in both groups. The clinical pregnancy and implantation rate also were not different statistically. The mean age of patients was 39.59 yr. However they believed that their results may not necessarily apply to younger patients and they declare that G-CSF losses its effectiveness in the presence of a normally proliferating endometrium, or at least when it infused intrauterine, and systemic G-CSF has different effects from local G-CSF (41).

Either G-CSF mechanisms is activation of some immunological process that are responsible for implantation or it reacts via inducing inflammation, it seems that in patients with recurrent implantation failure who have good quality embryos and uterine cavity has no polyp, myoma, adenomyosis, or adhesion or any space occupying lesion or hydrosalpinx, G-CSF may increase pregnancy and implantation rates without serious side effects (42). It seems that G-CSF can initiates a beneficial cross talk between endometrium and developing embryo and it can improve implantation through rolling, apposition, adhesion and invasion (43). This is the same stage that activated migrating leukocyte transferring vascular endothelium, where G-CSF affects this process; it has to be figure out and proven.

## Conclusion

G-CSF may increase pregnancy rate and implantation rate in recurrent implantation failure patients.

## Note

***Registration ID in IRCT:*** ***RCT201108212576N5***
